# Efficacy and Safety of Pegylated Interferon Plus Ribavirin Therapy for Chronic Hepatitis C Genotype 6: A Meta-Analysis

**DOI:** 10.1371/journal.pone.0100128

**Published:** 2014-06-25

**Authors:** Xiwei Wang, Fen Liu, Fang Wei, Hong Ren, Huaidong Hu

**Affiliations:** 1 Department of Infectious Diseases, Institute for Viral Hepatitis, Key Laboratory of Molecular Biology for Infectious Diseases, The Second Affiliated Hospital of Chongqing Medical University, Chongqing, PR, China; Temple University School of Medicine, United States of America

## Abstract

**Background:**

Hepatitis C genotype 6 (HCV-6) is prevalent in Southeast Asia. Data on the efficacy of direct-acting antiviral agents in chronic HCV-6 patients is limited and pegylated interferon (Peg-IFN) plus ribavirin (RBV) combination therapy remains standard therapy for those patients.

**Aim:**

Meta-analysis was performed to assess the efficacy and safety of Peg-IFN plus RBV combination therapy for chronic HCV-6 patients.

**Methods:**

Relevant studies were found by database search through Medline, Embase, Web of Science and The Cochrane Library. All published clinical trials assessing the efficacy of Peg-IFN plus RBV combination therapy for chronic HCV-6 patients were included. Sustained virological response rate (SVR) was pooled. We performed additional meta-analyses to compare the SVR outcomes of 24 versus 48 weeks of treatment in four head-to-head trials. Another second meta-analysis was also conducted to compare the efficacy of combination Peg-IFN plus RBV therapy in HCV-6 versus HCV-1 patients.

**Results:**

Thirteen studies met the inclusion criteria. The pooled SVR of all single arms was 75% (95% CI: 0.68–0.81). The SVR of 24 weeks treatment was significantly lower than that at 48 weeks, with a risk difference of −14% (95% CI: −0.25 to −0.02, p = 0.02). However, when restricted to the patients with rapid virological response (RVR), there was no significant effect on SVR between these two treatment groups, with a risk difference of −1% (95% CI: −0.1 to 0.07, p = 0.67). The SVR in HCV-6 patients was significantly higher than that in HCV-1 patients, with a relative risk of 1.35 (95% CI: 1.16–1.57, p<0.001). Side effects were common, but rarely caused treatment discontinuation.

**Conclusions:**

The results of this meta-analysis suggest that Peg-IFN plus RBV is effective and safe for HCV-6 patients. Shortening treatment seems to be feasible in HCV-6 patients with RVR when tolerance to treatment is poor. However, this decision should be made cautiously.

## Introduction

HCV infection frequently causes liver cirrhosis and liver cancer. Almost 3% of the world population have chronic HCV infection, and over 350,000 people die annually due to advanced liver disease [1]. Until now, six primary genotypes and numerous subtypes have been classified based on HCV nucleotide sequences [2]. HCV genotypes have a varied geographic distribution around the world. The most common genotypes in Western Europe and North America are 1, 2, and 3 [3],[4]. HCV genotype 4 is mostly distributed in the Middle East and North Africa, while genotype 5 is limited to South Africa. Of the primary HCV genotypes, genotype 6 is one of the most prevalent in Southern China and Southeast Asia, contributing to almost 30% of all HCV-infected patients in these areas [5]. Among the subtypes of HCV-6, subtype 6a is the most geographically limited having been discovered only in Vietnam, Macau and Hong Kong, or emigrants from those countries [6].

Recent advances in the development of direct-acting antiviral agents (DAAs), which inhibit viral proteins and block the HCV lifecycle, have revolutionized HCV therapy [7]. DAAs can achieve higher antiviral responses when combined with pegylated interferon (Peg-IFN) plus ribavirin (RBV) [8], [9]. They also have the potential to eradicate HCV without IFN [10]. In 2011, boceprevir and telaprevir received FDA approval for use in HCV-1 patients, and were the first approved DAAs for anti-HCV therapy [11]. Subsequently, a number of additional DAAs are being tested in ongoing clinical trials, and two are now commercially available. However, in many countries, DAAs are still not available or are too expensive for general use. As a result, in those places, a combination of Peg-IFN with RBV remains the standard therapy for chronic hepatitis C [12].

The HCV genotype is a crucial predictor of anti-viral therapeutic response, and is also important in determining treatment duration [13]. Patients with HCV-1 have a much lower sustained virological response (SVR) rate of only ∼40–50% with Peg-IFN plus RBV combination therapy. In contrast, HCV-2 is considered an “easy to treat” genotype compared to HCV-1 [14]. Furthermore, many recent studies have indicated that HCV-2 can achieve higher SVR than HCV-3 [15], [16]. Consequently, the package inserts state that HCV-1 patients should be treated for 48 weeks with Peg-IFN plus RBV combination therapy, whereas for HCV-2 patients, 24 weeks was recommended [12], [17]. However, the data on expected response rates and optimal treatment duration for HCV genotype 6 (HCV-6) are limited compared to what is known for genotypes 1–3.

Meta-analysis is a useful quantitative approach to combine the results from multiple studies, especially when the results of the studies are not consistent. Although the efficacy and safety of Peg-IFN and RBV combination therapy in HCV-6 patients have been evaluated by others through a systematic review [2], no comprehensive meta-analyses of clinical trial data were reported. The aim of the present study was to conduct a meta-analysis of trials to assess the efficacy and safety of Peg-IFN and RBV treatment in chronic HCV-6 patients.

## Methods

### Eligibility

Our review included eligible clinical trials that assessed the efficacy of combination therapy with Peg-IFN and RBV in chronic HCV-6 patients. For inclusion, published articles or abstracts had to: (a) have a protocol with an adequate course of treatment (24 or 48 weeks); (b) provide information on a primary outcome of interest clearly defined as sustained virological response (SVR), which was defined as undetectable HCV RNA at least 24 weeks after the end of treatment; (c) focus on treatment-naïve adult patients; (d) be reported in English. We excluded the studies referring to patients with other HCV genotypes, hepatitis B virus or human immune deficiency virus co-infections, other liver diseases, hemophilia, chronic renal failure, liver decompensation, liver transplantation, liver cancer, and psychosis. The studies that had less than 10 HCV-6 patients were also excluded. When there were multiple publications from the same population, only data from the most recent and complete ones were selected. Moreover, we contacted the authors if further detailed data was required.

### Search strategy

Searches of Medline, Embase, Web of Science and The Cochrane Library, from inception to December 2013, were conducted by two investigators (XWW and FL). The following keywords were searched in combination of MeSH terms and text words: “hepatitis C”, “genotype”, “peg-interferon” and “ribavirin”. To locate additional studies, relevant reference lists, journals and abstracts of two international meetings in the areas of liver disease (AASLD and EASL) were also searched. Furthermore, we corresponded with authors for more information when necessary.

### Study selection and data extraction

Titles and abstracts of potentially eligible publications were screened by two investigators (XWW and FL). We retrieved and independently reviewed all the trials for possible inclusion. Conflicting opinions were resolved by consensus between the two investigators. When required, the corresponding author (HDH) provided arbitration.

Using a data collection form, each selected study was abstracted independently and in duplicate by the two reviewers (XWW and FL). The following information was extracted: year of publication, study design, study population, number of participants per study arm, baseline patient characteristics, treatments received, duration of follow up, and virological responses. Where possible, data on adverse events were extracted as well. Any disagreement between the reviewers was resolved as mentioned above.

### Endpoints

Endpoints were defined prior to the initiation of the study. To estimate the efficacy of Peg-IFN plus RBV treatment in the selected trials, the SVR rate (percentage of patients with undetectable HCV RNA at least 24 weeks after the end of treatment) was defined as the primary outcome. The secondary outcomes were the rates of rapid virological response (RVR, undetectable HCV RNA at week 4), treatment discontinuation and adverse events. Differences in the limits of HCV RNA detectability among the studies are shown in Table 1.

**Table 1:** Characteristics of included trails.

Study	Publication	Study	Study	Genotype	Regimen	Treatment	Sample	Mean	Males	Of	Lower detection	SVR	RVR	SVR among	Relapse	Treatment
	type	design				Duration	size	age (years)	No.(%)	cirrhosis	limit of	No.(%)	No.(%)	RVR patients	No.(%)	discontinuation due to AEs
						(weeks)				No.(%)	HCV-RNA			No.(%)		No.(%)
Nguyen et al	Full	Nonrandomized	Asian	6	Peg-IFN α-2a 180 µg/week + RBV 1,000–1,200 mg/day or	24	23	49±10	16 (69.6)	0	50 IU/mL or	9 (39)	NR	NR	6 (35)	NR
2008		trail	American	6	Peg-IFN α-2b 80–150 µg/week + RBV 800–1,200 mg/day	48	12	50±10	7 (58.3)	0	160 copies/mL	9 (75)	NR	NR	2 (17)	NR
Fung et al	Full	Nonrandomized	Chinese	1	Peg-IFN α-2a 180 µg/week + RBV 1,000–1,200 mg/day or	48	21	52 (30–63)	12 (57)	3 (14)	NR	11 (52)	NR	NR	NR	-
2008		trail		6	PEG IFN- α-2b 1.5 µg/kg + RBV 800–1,200 mg/day	48	21	49.5 (14–64)	11 (52)	6 (29)		18 (86)	NR	NR	NR	6 (29)
Lam et al	Full	Randomized controlled	Asian descent	6	Peg-IFN α-2a 180 µg/week + RBV 800–1,200 mg/day	24	27	49.6±12.4	13 (48)	0	50 IU/mL	19 (70)	17 (85)	14 (82)	NR	6 (22)
2010		trail		6		48	33	52.8±8.0	15 (46)	0		26 (79)	12 (63)	10 (83)	NR	6 (18)
Nguyen et al*	Full	Nonrandomized	Asian	1	Peg-IFN α-2a 180 µg/week + RBV 800–1,200 mg/day or	48	70	50±9.7	51 (73)	8 (11)	50 IU/mL or	25 (49)	NR	NR	NR	NR
2010		trail	American	6	Peg-IFN α-2b 80–150 µg/week + RBV 800–1,200 mg/day	24	27	49.4±12.0	15 (56)	2 (7)	160 copies/mL	17 (63)	NR	NR	NR	NR
						48	34	49.4±10.8	19 (56)	3 (9)		34 (74)	NR	NR	NR	NR
Tsang et al	Full	Nonrandomized	Chinese	1	Peg-IFN α-2a 180 µg/week + RBV 1,000–1,200 mg/day or	48	70	48 (18–64)	44 (63)	0	50 IU/mL	40 (57.1)	NR	NR	NR	-
2010		trail		6	PEG IFN- α-2b 1.5 µg/kg + RBV 800–1,200 mg/day	48	70	50 (28–75)	23 (33)	0		53 (75.7)	NR	NR	NR	17 (24.3)
Zhou et al*	Full	Nonrandomized	Chinese	1	Peg-IFN α-2a 180 µg/week + RBV 1,000–1,200 mg/day or	48	39	15 (38.5) #	22 (56.4)	0	NR	23 (59.0)	-	-	-	NR
2011		trail		6	PEG IFN- α-2b 1.5 µg/kg + RBV 800–1,200 mg/day	24	22	19 (86.4) #	14 (63.6)	0		18 (81.8)	15 (83.3)	13 (86.7)	2 (10.0)	NR
Qing-Xian et al	Abstract	Nonrandomized	Chinese	6	pegylated interferon and ribavirin	48	84	NR	NR	0	NR	74(88.1)	60(71.4)	NR	NR	NR
2011		trail														
Tangkijvanich* et al	Full	Nonrandomized	Thai	1	Peg-IFN α-2a 180 µg/week + RBV 1,000–1,200 mg/day	48	16	46.4±12.5	9 (56.3)	0	NR	10 (62.5)	-	-	-	-
2012		trail		6		RGT&	34	41.2±8.4	23 (67.6)	0		26 (76.5)	25 (73.5)	22(88)	3 (12)	0
Shao et al	Abstract	Nonrandomized trail	Chinese	6	pegylated interferon and ribavirin	48	28	NR	NR	0		26 (92.8)	26 (92.8)	NR	NR	NR
2012																
Mauss et al*	Full	Nonrandomized	Asian	6	Peg-IFN α-2a 180 µgweek + RBV 1,000–1,200 mg/day	47 (47–48)	27	47 (37–52)	17 (63)	0	50 IU/mL	16 (59)	5 (45)	NR	4 (15)	3 (11)
2012		trail														
Thu Thuy et al	Full	Randomized controlled	Vietnamese	6	Peg-IFN α-2a 180 mg/week +RBV 15mg/kg/day	24	35	46.82±7.2	22 (62.85)	0	100 copies/ml	21 (60)	28 (80)	21 (75)	2 (7)	0
2012		trail		6		48	70	48.57±8.4	43 (61.42)	0		50 (71)	57 (81)	49 (86)	3 (5)	0
Seto et al	Full	Nonrandomized	Chinese	6	Peg-IFN α-2a 180 µg/week + RBV 1,000–1,200 mg/day or	48	60	49 (14–71)	41 (68.3)	3 (5)	NR	55 (91.7)	NR	NR	NR	NR
2013		trail			PEG IFN- α-2b 1.5 µg/kg + RBV 800–1,200 mg/day											
Qing-Xian et al	Abstract	Randomized controlled	Chinese	6	Peg-IFN α-2a 180 µg/week + RBV 800–1,200 mg/day	24	242	NR	NR	NR	15 IU/mL	NR	152 (62.8)	56 (93.3)	NR	NR
2013		trail		6		48		NR	NR	NR		NR		52 (94.5)	NR	NR

# Age<40 years n (%); NR: Not Reported; RGT: Respond-Guided therapy; AEs: adverse events.

*Including HCV-2/3 and HCV-6 groups. Only the data on HCV-6 groups were included in the current meta-analysis.

### Statistical Analysis

Primary and secondary outcomes were evaluated by calculating point estimates and 95% confidence intervals (CIs) and p values. We used a Freeman-Tukey type arcsine square root transformation [18] to stabilize the variance of raw proportions (r/n), and then pooled the proportions by a DerSimonian–Laird random-effects model [19], [20]. As errors may occur when evaluating heterogeneity in pooled proportions, we calculated the *I* ^2^value when required [21]. To assess sources of potential bias, sensitivity analyses were conducted for included studies where required. For some direct comparison studies, we calculated the risk difference of SVR between 24 and 48 weeks treatment for HCV-6 patients. In some trials, we included head-to-head comparison of antiviral therapeutic efficacy in HCV-1 versus HCV-6. A second meta-analysis was performed to pool the relative risk of SVR. Furthermore, we constructed funnel plots for both primary and secondary outcomes, together with Egger's regression asymmetry test and Begg's rank correlation test [22], [23], to assess potential publication bias. All analyses were conducted by Stata (version 12.0) and R (version 3.0.2) software, with a P<0.05 considered significant.

## Results

**Figure 1:**
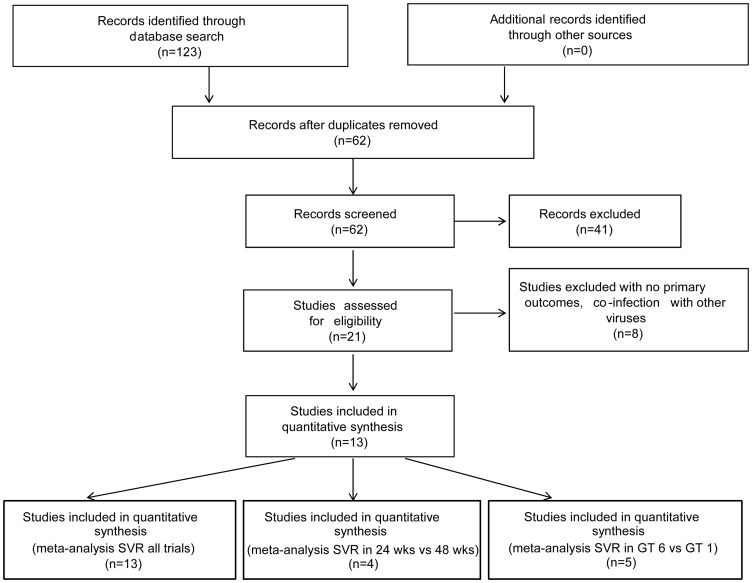
Study selection procedure.

We initially identified 123 citations by manual and electronic database searches. After removing 61 duplicate citations, 62 remained. We screened the titles and abstracts of remaining citations and found 21 potentially eligible trials. Eight potential trials were excluded because those studies included co-infected patients, and could not provide sufficient data on primary outcomes. After parsing, 13 studies were included in this meta-analysis [24]–[36]. Figure 1 shows a diagram of the process of study selection.

Among the 13 eligible trials, 10 were published as full-texts, while 3 were abstracts [24], [29], [36]. The characteristics of included studies are shown in Table 1. In total, the pooled number of HCV-6 patients in these studies was 849 participants who were mostly Asian or Asian descent.

**Figure 2:**
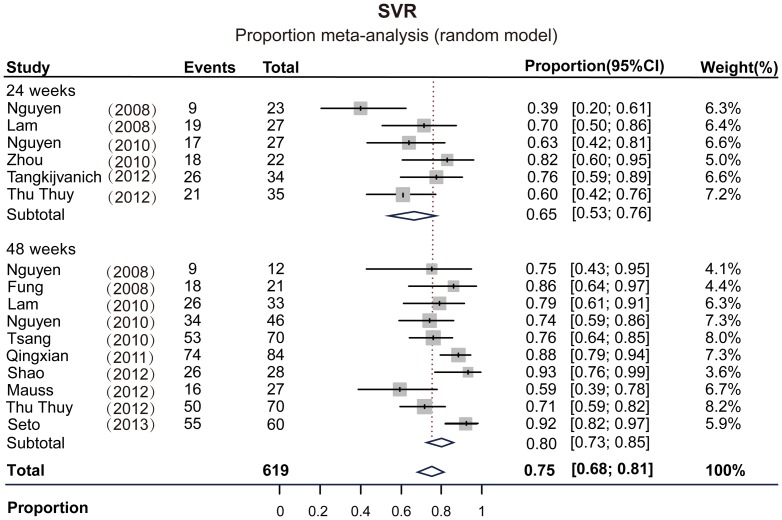
Proportion meta-analysis of SVR rates in all eligible study arms in HCV-6 patients.

**Figure 3:**
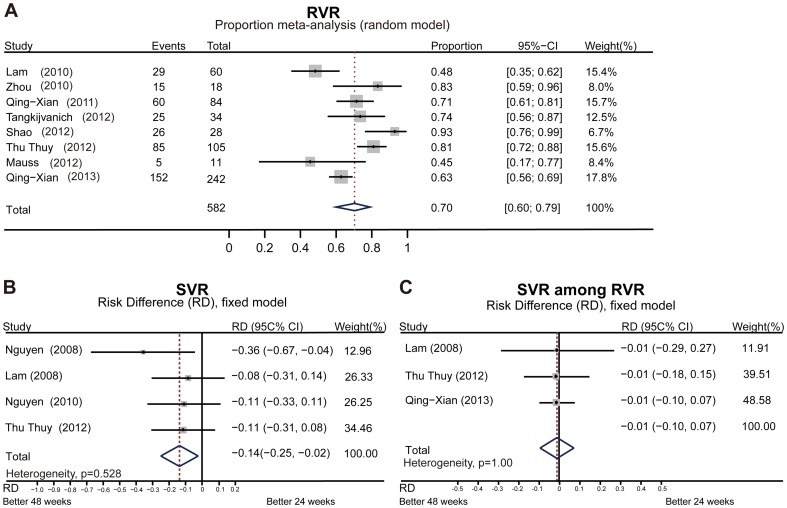
Pooled proportion of RVR and evaluated the effect of RVR on SVR. (A) Pooled proportion of RVR in all eligible study arms. (B) Overall analysis of studies head-to-head directly assessing 24-week versus 48-week treatment for HCV-6 patients. (C) Sensitivity analysis restricted to studies evaluating 24-week versus 48-week treatment among RVR patients.

All 13 trials reported SVR data. HCV treatment was consisted of Peg-IFN plus weight-based RBV in 11 trials. The SVR for HCV-6 patients ranged from 39% to 92.8% in these trials. As shown in Figure 2, the pooled SVR across all study arms was 75% (95% CI: 0.68–0.81, *I* ^2^ = 65.9%), while the pooled SVR for 24 and 48 weeks treatment were 65% (95% CI: 0.53–0.76, *I* ^2^ = 56.2%) and 80% (95% CI: 0.73–0.85 *I* ^2^ = 59.3%), respectively. The RVR for HCV-6 patients ranged from 48% to 93%, with pooled rate of 70% (95% CI: 0.60–0.79, *I* ^2^ = 77.9%, Figure.3A). Information on adverse events and treatment discontinuations due to adverse events were shown in Table 2.

**Table 2:** Proportion meta-analysis of Safety Outcomes by Random Effects Model.

Adverse Events	24 Week	48 Week	p-value
	Rate (95%CI)	Rate (95%CI)	
Discontinuation due to AEs	0.07(0.01–0.21)	0.15(0.07–0.27)	0.18
Relapse	0.18(0.03–0.30)	0.11(0.06–0.17)	0.16
IFN reduction	0.07(0.01–0.26)	0.16(0.09–0.25)	0.10
RBV reduction	0.18(0.02–0.35)	0.29(0.20–0.41)	0.18
Use of erythropoietin	0.15(0.06–0.34)	0.27(0.05–0.75)	0.13
Thrombocytopenia	0.02(0.01–0.09)	0.02(0.01–0.05)	1.00
Anemia	0.30(0.17–0.48)	0.44(0.28–0.61)	0.02
Depression	0.14(0.03–0.24)	0.11(0.03–0.35)	0.28
Insomnia	0.41(0.16–0.71)	0.32(0.12–0.63)	0.20
WBC<1500 and/or ANC<750 cells/mm 3	0.13(0.05–0.30)	0.17(0.10–0.28)	0.29
Hyperthyroidism	0.03(0.01–0.06)	0.04(0.02–0.08)	0.86
Alopecia	0.18(0.01–0.45)	0.22(0.05–0.61)	0.60

Four studies included head-to-head comparisons of 24 week versus 48 week treatment with Peg-IFN plus RBV for HCV-6 patients [27], [31], [32], [34]. We conducted a second meta-analysis of these four studies, and found that the SVR rate for 24 weeks of treatment was significantly lower than that for 48 weeks. The risk difference was −14% (95% CI: −0.25 to −0.02, p = 0.019, Figure.3B). However, after performing a sensitivity analysis restricted to three studies that evaluated 24- versus 48-week treatments among RVR patients [24], [27], [34], we found no significant effect on the SVR rate after 24 weeks of treatment. The risk difference was −1% (95% CI: −0.1 to 0.07, p = 0.765, Figure. 3C).

**Figure 4:**
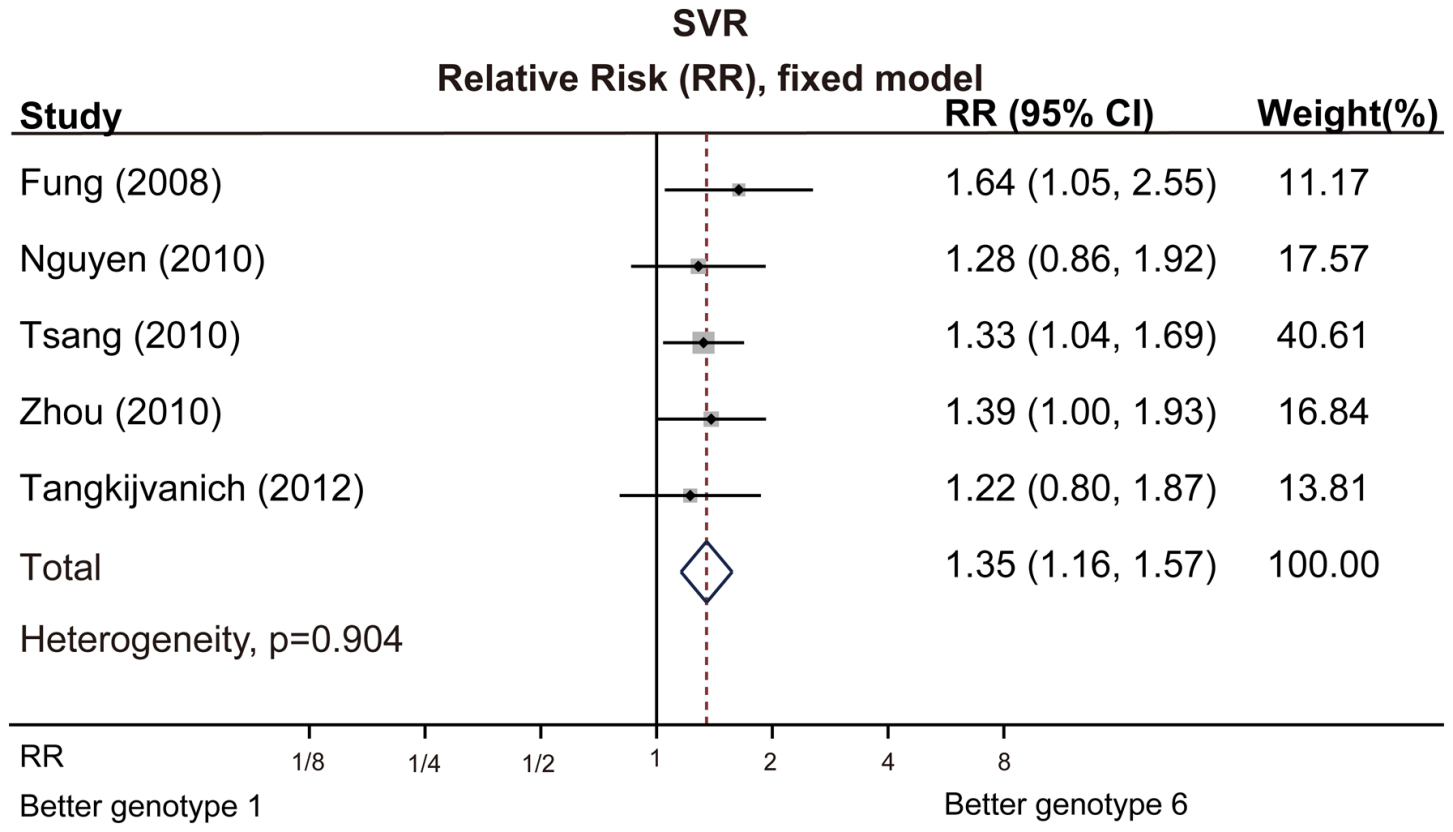
Meta-analysis of SVR rates in HCV-1 versus HCV-6 patients.

Five trials were direct comparisons of antiviral therapy in HCV-6 versus HCV-1 patients [25], [26],[28],[31],[35]. Among these trials, we found that SVR rate in HCV-6 patients was higher than that in HCV-1 patients: 75.9% vs 55.3%, with a relative risk of 1.35 (95% CI: 1.16–1.57, p<0.001) (Figure. 4). No significant publication bias was found by Egger's funnel plot asymmetry test, and Begg's rank correlation test (Figure. S1, S2, S3, S4).

## Discussion

To our knowledge, no studies to date have been conducted to evaluate the efficacy of boceprevir and telaprevir, the most recently approved DAAs, in HCV-6 patients. In recent phase II/III trials, simeprevir [37] and sofosbuvir [38] were shown to have clinical benefit in HCV-6 patients, although the sample sizes were small. DAAs seem to be effective in this group, but the data are too limited to make a general recommendation [39], [40]. In addition, DAAs are not available in most countries in Southeast Asia because of socio-economic and other obstacles [41]. Until these issues are resolved, Peg-IFN plus RBV combination therapy remains appropriate for patients with HCV-6. However, most available data has focused on treatment of genotypes 1, 2 and 3, which are prevalent in Western Europe and North America, where the majority of multicenter trials have been performed. Optimal treatment duration and expected virological response of Peg-IFN plus RBV combination therapy in HCV-6 patients has not yet been determined. Previous results comparing 24 and 48 weeks of treatment remain conflicting. Nguyen et al., in a retrospective study, found that HCV-6 patients treated for 48 weeks had significantly higher SVR rates than those treated for 24 weeks. Nevertheless, in two randomized controlled studies published recently, similar rates of SVR were noted following 24 weeks and 48 weeks of therapy [27], [34]. This may be due to small sample sizes that made meaningful differences difficult to discern. Although it has been suggested that the response to antiviral therapy in HCV-6 patients is higher than that in HCV-1 patients, most related studies included small sample sizes, and failed to give effective recommendations [25],[26],[28],[31],[35]. Furthermore, one recent retrospective study also found that SVR in HCV-1 patients and HCV-6 patients was not significantly different [42].

In the present meta-analysis, we assessed efficacy in the light of virological outcomes. As for the safety, side effects and treatment discontinuation of peg-IFR and RBV were examined as well. Furthermore, a second meta-analysis of suitable trials was conducted to compare 24 weeks versus 48 weeks treatment in HCV-6 patients. For a more comprehensive understanding of the efficacy of this treatment strategy in HCV-6 patients, we also evaluated SVR for HCV-6 and HCV-1 patients in some head-to-head comparison trials. Our analyses indicate that Peg-IFN plus RBV was effective in the majority of HCV-6 patients, and efficacy was higher than that for genotype 1. Side effects were common, and consisted mostly of hematologic and dermatologic events which rarely caused treatment discontinuation.

The optimal duration of treatment in HCV-6 patients has been studied previously [27], [31], [32], [34]. In the present study we performed a second meta-analysis to assess the efficacy of Peg-IFN plus RBV in HCV-6 patients for 24 versus 48 weeks treatment. Pooled data suggested that 48 weeks of treatment may be more effective in inducing SVR than 24 weeks of treatment. Nevertheless, when restricted to RVR patients in head-to-head treatments at 24 weeks compared to 48 weeks, no statistical differences were found in SVR [24], [27], [34]. These results indicated that shortened treatment may be appropriate in RVR group. A previous retrospective cohort study reported higher SVR after 48 weeks of treatment. This discrepancy may have been due to the small sample size and lack of intention-to-treat analysis [32]. Although the present meta-analysis showed that 24 weeks of therapy achieved similar efficacy compared to 48 weeks of treatment, clinicians should consider shortening treatment in HCV-6 patients with RVR cautiously, as the data from this meta-analysis are not sufficient to recommend treatment duration for all HCV-6 patients. Thus, we recommend larger randomized controlled trials to define the optimal treatment duration in such patients. A recent study had found that the IL28B rs8099917 TT genotype was significantly related to an increased SVR rate compared to the TG genotype [30]. These results suggest that IL28B could be another important potential predictor, and deserves further study.

There are limitations to the present study. Although our study has included all related work in one recent systematic review, the number of trials that met the inclusion criteria was small. Given these limited trials, the pooled data represent various study designs, and only included three randomized controlled trials. In addition, although the efficacy of Peg-IFN and RBV may vary depending on the dose schedules, and types of treatment regimen, we were not able to perform sensitivity analyses on these parameters due to limited data. Besides, limited information on other potential predictors of SVR such as early virological response, male sex, increasing age, AST/ALT ratio and viral load, also prevented quantitative analysis. Although most trials only included treatment of naive patients, some trials included a few treated patients. Furthermore, three included trials were only available as abstracts. However, these trials met all the inclusion criteria, and could provide data on the outcomes of interest. We therefore included these studies in our meta-analysis here. Despite these limitations, the present meta-analysis study provides the most comprehensive summary of evidence to date on the effectiveness of Peg-IFN plus RBV in treating HCV-6 patients.

In conclusion, based on the available data, our results indicate that Peg-IFN plus RBV is effective for patients with HCV-6, and the efficacy was superior compared to patients with HCV-1. With this treatment schedule, about three-quarters of patients are expected to achieve SVR. Furthermore, shortening treatment seems to be feasible in HCV-6 patients with RVR when tolerance to treatment is poor, but the decision should be make cautiously.

## Supporting Information

Figure S1 **Publication bias tests for proportion meta-analysis of SVR in 24-week (A) or 48-week (B) treatment arms.**
(TIF)

Figure S2 **Publication bias test for proportion meta-analysis of RVR in all eligible study arms.**
(TIF)

Figure S3 **Publication bias tests when evaluating the effect of RVR on SVR.** (A) Publication bias test for overall analysis of SVR in trials comparing the efficacy of 24- versus 48- week treatment directly. (B) Publication bias test for the sensitivity analysis of SVR among RVR patients after 24- versus 48- week treatment.
(TIF)

Figure S4 **Publication bias test for SVR in trials directly comparing antiviral therapy in HCV-6 versus HCV-1 patients.**
(TIF)

Checklist S1 **The PRISMA checklist.**
(DOCX)
