# Supplementary figures and images for: Efficacy and Safety of Pegylated Interferon Plus Ribavirin Therapy for Chronic Hepatitis C Genotype 6: A Meta-Analysis
Source: PLoS One. 2014 Jun 25;9(6):e100128. doi: 10.1371/journal.pone.0100128 (PMC4070902; doi:10.1371/journal.pone.0100128)

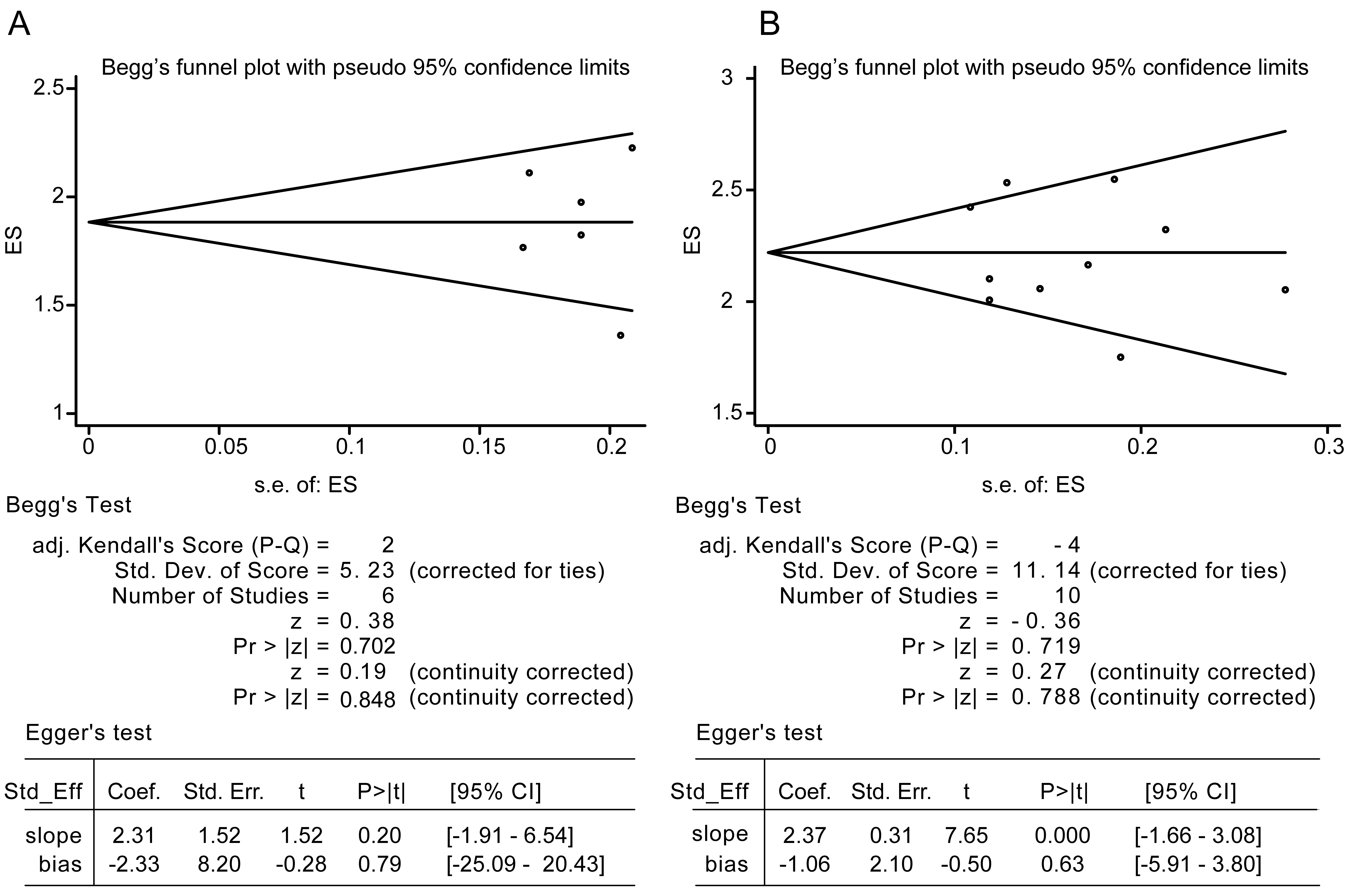

Supplement: Figure S1 — Publication bias tests for proportion meta-analysis of SVR in 24-week (A) or 48-week (B) treatment arms. (TIF) [file pone.0100128.s001.tif]

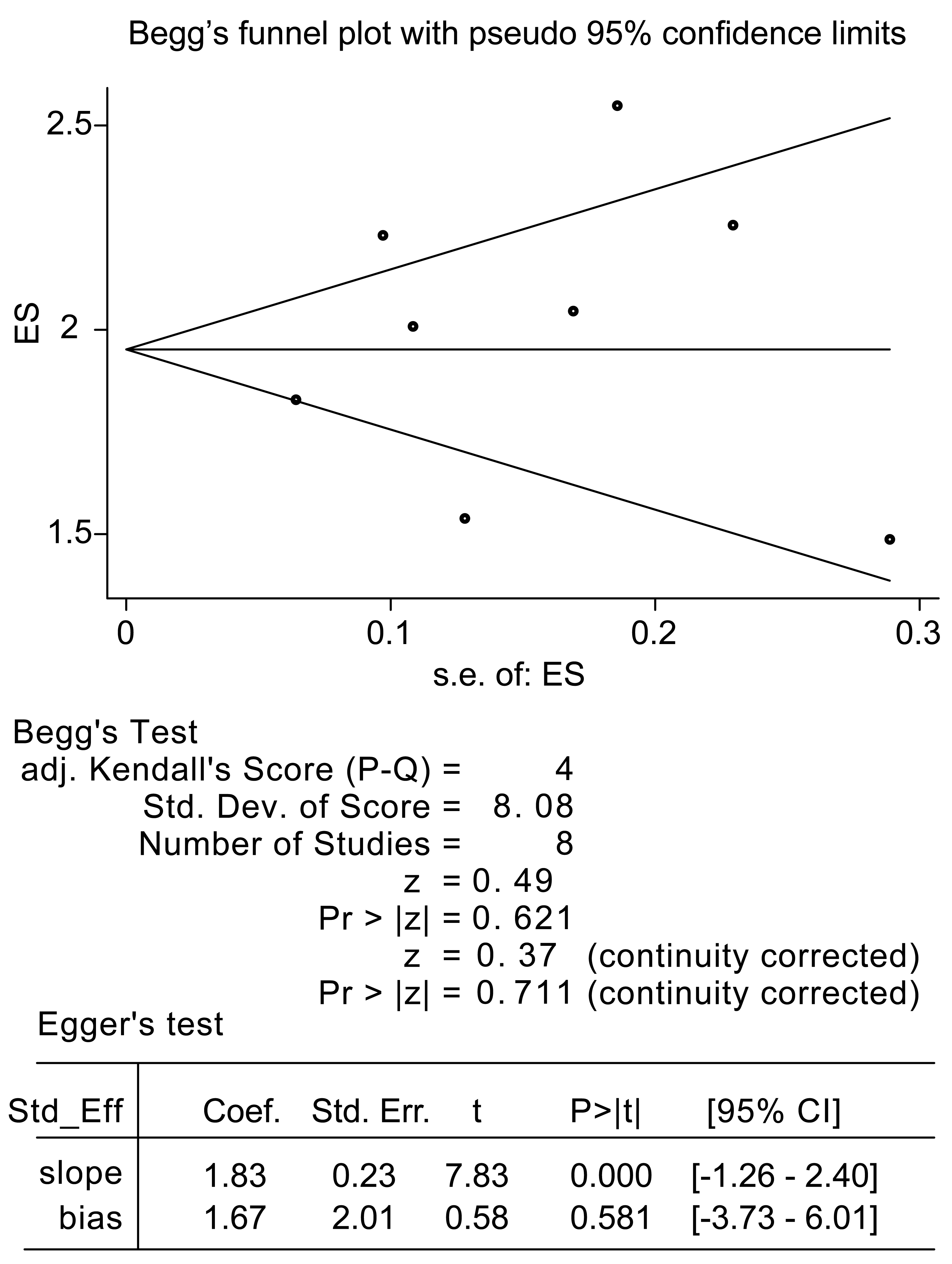

Supplement: Figure S2 — Publication bias test for proportion meta-analysis of RVR in all eligible study arms. (TIF) [file pone.0100128.s002.tif]

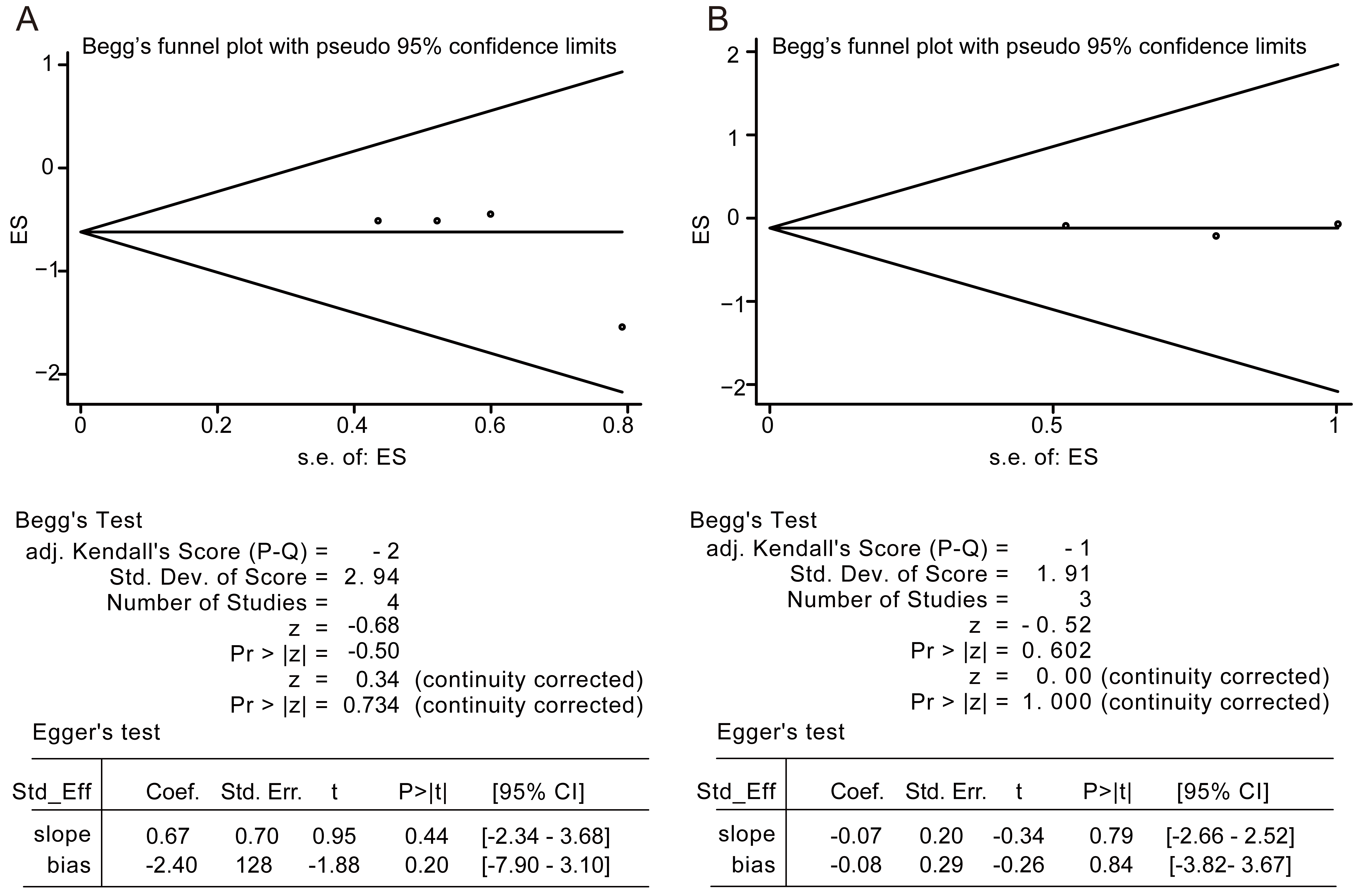

Supplement: Figure S3 — Publication bias tests when evaluating the effect of RVR on SVR. (A) Publication bias test for overall analysis of SVR in trials comparing the efficacy of 24- versus 48- week treatment directly. (B) Publication bias test for the sensitivity analysis of SVR among RVR patients after 24- versus 48- week treatment. (TIF) [file pone.0100128.s003.tif]

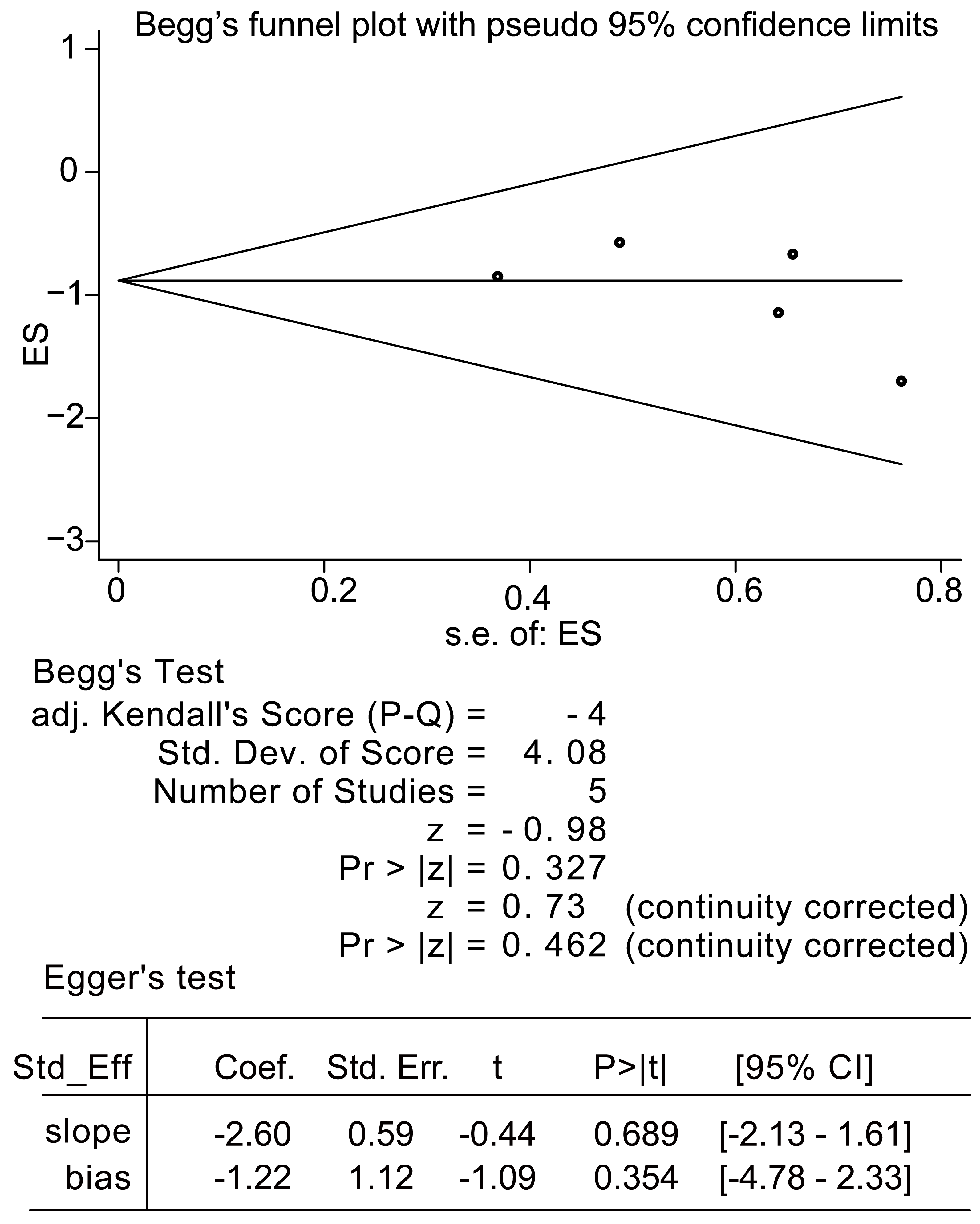

Supplement: Figure S4 — Publication bias test for SVR in trials directly comparing antiviral therapy in HCV-6 versus HCV-1 patients. (TIF) [file pone.0100128.s004.tif]
